# Lung cancer in never smokers: Tumor immunology and challenges for immunotherapy

**DOI:** 10.3389/fimmu.2022.984349

**Published:** 2022-08-24

**Authors:** Viviane Teixeira L. de Alencar, Amanda B. Figueiredo, Marcelo Corassa, Kenneth J. Gollob, Vladmir C. Cordeiro de Lima

**Affiliations:** ^1^ Medical Oncology Department, Grupo Carinho de Clínicas Oncológicas, São José dos Campos, Brazil; ^2^ Translational Immuno-oncology Laboratory, Albert Einstein Research and Education Center, Hospital Israelita Albert Einstein, São Paulo, Brazil; ^3^ Center for Research in Immuno-oncology (CRIO), Hospital Israelita Albert Einstein, São Paulo, Brazil; ^4^ Medical Oncology Department, A C Camargo Cancer Center, São Paulo, Brazil

**Keywords:** nsclc, never smokers, immunotherapy, immune checkpoint inhibitor, tumor microenvironment

## Abstract

Lung cancer is the second most common and the most lethal malignancy worldwide. It is estimated that lung cancer in never smokers (LCINS) accounts for 10-25% of cases, and its incidence is increasing according to recent data, although the reasons remain unclear. If considered alone, LCINS is the 7th most common cause of cancer death. These tumors occur more commonly in younger patients and females. LCINS tend to have a better prognosis, possibly due to a higher chance of bearing an actionable driver mutation, making them amenable to targeted therapy. Notwithstanding, these tumors respond poorly to immune checkpoint inhibitors (ICI). There are several putative explanations for the poor response to immunotherapy: low immunogenicity due to low tumor mutation burden and hence low MANA (mutation-associated neo-antigen) load, constitutive PD-L1 expression in response to driver mutated protein signaling, high expression of immunosuppressive factors by tumors cells (like CD39 and TGF-beta), non-permissive immune TME (tumor microenvironment), abnormal metabolism of amino acids and glucose, and impaired TLS (Tertiary Lymphoid Structures) organization. Finally, there is an increasing concern of offering ICI as first line therapy to these patients owing to several reports of severe toxicity when TKIs (tyrosine kinase inhibitors) are administered sequentially after ICI. Understanding the biology behind the immune response against these tumors is crucial to the development of better therapeutic strategies.

## Introduction

Lung cancer is the second most common malignancy diagnosed annually worldwide, close behind breast cancer, with more than 2.2 million new cases each year. It is also responsible for approximately 1.8 million deaths a year, representing the leading cause of cancer-related death (1).

Tobacco smoking is the main risk factor associated with this malignancy and is responsible for about two-thirds of lung cancer deaths (1). Smokers have a 10-20-fold increase in lung cancer risk when compared to never-smokers (2). Current anti-tobacco policies have reduced this habit globally, especially in high income countries (HIC), with over 80% of current smokers ≥ 15 years-old living in low to middle income countries (LMIC) (3). In spite of that, it is expected that lung cancer incidence will increase worldwide by 64.4% by 2040, for reasons yet to be defined (4). Apart from known environmental risk factors, recent studies have investigated the potential carcinogenic effect of genetic alterations in the development and proliferation of lung cancer cells. In fact, lung cancer in smokers and never-smokers have different molecular profiles, as well as distinct tumor microenvironments (TME) (5) which impact the susceptibility to novel treatments, such as targeted-therapies and immunotherapy.

The aim of this review is to discuss the different aspects of lung cancer in never smokers, with a focus on tumor immunology and the current evidence on the use of immunotherapy for this subgroup of patients.

## Epidemiology and risk factors

### Lung cancer in never smokers

Lung cancer in never smokers (LCINS) corresponds to 10-25% (6) of all lung cancer cases, with an even higher proportion, up to 65%, in women from Asia and Middle Eastern countries (2). It is more common in women, with adenocarcinoma histology (7) and tends to be diagnosed at more advanced stages, possibly due to a lack of suspicion in patients without history of tobacco use (8). However, the age at diagnosis varies according to geographic location. In Asia, LCINS tends to occur at a younger age (9, 10), while in the United States and Europe it appears to be diagnosed at the same or older ages than in smokers (11, 12). Nevertheless, LCINS has a better prognosis than tobacco-related lung cancer, which may be related to a higher frequency of mutations in actionable driver genes (7) and a different composition of immune cells in the tumor microenvironment, which are related to better clinical outcomes (13). In fact, never smokers with lung cancer appear to have a better survival independent of stage, treatment and other factors (14).

In spite of that, this malignancy is still an important public health issue, as it is considered the 7th most common cause of cancer death, ahead of cervical, pancreatic and prostate cancer (14).

There are several risk factors that have been associated with lung cancer development apart from tobacco smoking, although none were found exclusively among never-smokers, suggesting a heterogeneous carcinogenic process (15). The most commonly associated factors are: outdoor air pollution, domestic fuel smoke (wood burning), chronic pulmonary diseases, occupational exposure to carcinogenic chemicals (silica, arsenic, chromium, cadmium, nickel), ionizing radiation (such as residential radon), alcohol consumption (5, 14) and family history/genetics.

### Environmental tobacco exposure (passive smoking, secondhand smoke)

Several studies worldwide have associated secondhand smoke to the risk of lung cancer. An American multicenter study found a 30% increase in the risk of lung cancer in never-smokers with a smoking spouse (adjusted OR to all types of lung carcinoma = 1.29, p < 0.05) (16). Although passive smoking increases the risk of developing lung cancer by 20-24%, it is estimated that only 16-24% of lung cancers in never smokers are attributable to this cause (17). It has been considered a weak carcinogen compared to active smoking, and genomic studies have failed to detect smoking mutational signatures, such as SBS4 (Single Base Substitution Signature 4), above the threshold of 15% among these tumors, suggesting alternative carcinogenic processes (18).

### Radon

Radon is a radioactive gas produced from the decay of uranium present in soil and rocks (10.1200/JCO.2006.06.8015.). It was defined as a human carcinogen by the International Agency for Research on Cancer (IARC) in 1988 and epidemiological studies (19–21) have successfully correlated radon exposure to lung cancer both in miner workers and other environmentally exposed populations  (15). It is considered the second most important risk factor for the development of lung cancer after tobacco and the main risk factor amongst never smokers (22).

### Occupational exposure to carcinogenic chemicals or ionizing radiation

Occupational exposure to different kinds of carcinogenic chemicals or ionizing radiation is thought to be associated with 5-10% of lung cancer cases, and asbestos exposure is currently the most important factor, with a 5-fold increased risk of lung cancer (15). Other related carcinogens are arsenic exposure, with a lung cancer odds ratio up to 8.9 depending on the water concentration (23), chromium, nickel (24), silica (25) and solvents, paints or thinners (OR = 2.8) (26).

### Air pollution

Outdoor air pollution is a mixture of various pollutants originating from natural and anthropogenic sources, such as transportation, industrial activity, power generation, among others (27). The carcinogenic effects of outdoor air pollution has been observed in various studies, such as epidemiological research, experimental studies in animals and in cohort and case-control studies from different continents (27). In 2013, IARC classified outdoor air pollution and particulate matter from outdoor air pollution as carcinogenic to humans (27).

### Family history/genetics

More recently, studies have also investigated the possibility of inherited predisposition to lung cancer, and it is estimated that 5.8% of lung cancer patients present germline mutations in hereditary cancer genes (28). Familial aggregation studies have estimated an increased risk of developing lung cancer of up to 4-fold according to family history of lung cancer in first degree relatives and respective age at diagnosis (29, 30). Genome-wide association studies have suggested a susceptibility locus for lung cancer at 15q25.1, which contains nicotinic acetylcholine receptors genes (31–33). Hung et al. (33) observed an increased risk of lung cancer in non-smokers associated to the chromosome 15q25 locus, which suggested a different disease mechanism, not related to tobacco addiction, since it was not associated to smoking-related head and neck cancers. Other susceptibility loci, such as chromosomes 6q (34), 13q31.3 (*GPC5*), have also been described (35). Many studies have identified specific germline pathogenic mutations in lung cancer patients that could be associated with its development, such as *ATM, EGFR, TP53, BRCA, PARK2, YAP1* and *HER-2*. These alterations, however, are rare, and most still lack evidence of the association to the development of this specific malignancy (36–39).

## Carcinogenesis and molecular characteristics of lung cancer in never smokers

The tumorigenesis of LCINS is poorly understood, but genomic studies suggest that the pathways activated during LCINS carcinogenesis are different from tobacco-related lung cancer. Tobacco exposure is associated with genome-wide C to A transversions. Lung cancer in never smokers has a low fraction of C to A transversions, and additionally these tumors are enriched in mutations in *EGFR* and *PIK3CA*, in-frame insertions in *EGFR* and *ERBB2*, and frameshift indels in *RB1* (40, 41).

One of the first studies that directly compared lung cancer from smokers and never smokers, performed whole genome sequencing (WGS) in tumor and normal tissue samples obtained from 17 lung cancer patients, five of whom were never smokers. As demonstrated previously by Ding et al. (2009) (41), these tumors had a 10-fold lower mutation burden compared to tumors from smokers (0.6 mut/Mb x 10.5mut/Mb), as well as *EGFR* mutations, and *ALK* and *ROS1* fusions (42).

Chen et al. (43) performed a proteogenomics analysis of patient-paired tumor and normal adjacent tissue of 103 patients with treatment-naive lung cancer from Taiwan. In this cohort, 83% of patients were non-smokers, 89% were adenocarcinoma, 80% were stage IA or IB and 58% were female. Genetic mutations were most commonly found in *EGFR* (85%), *TP53* (33%) and *RBM10* (20%). *KRAS* and *ATM* alterations were more notable among patients with a history of tobacco use. They identified over 23,000 nonsynonymous somatic single nucleotide variants (SNVs), which had different proportions when compared to the TCGA (The Cancer Genome Atlas) cohort, mainly composed of smokers. Non-smokers in both cohorts showed similar proportions of C>T transitions, while C>A transversions, which are smoking-related, were more common in the TCGA cohort. In the non-smoking cohort, the study also identified five mutational signatures, not observed in the TCGA cohort. The APOBEC mutational signature, with a high proportion of C>T transitions, was associated with a potential role in the development of lung cancer in female never-smoker patients (43).

More recently, 46 NSCLC samples from never (36 patients) or light smokers (<5 pack-years and >20 years smoking-free interval, 10 patients) were submitted to low-coverage WGS and whole exome sequencing (WES) in parallel with WES from blood. These tumors displayed low tumor mutational burden (TMB) (0.8 mut/Mb) and less somatic copy number alterations (sCNAs) than tumors from smokers. Tumors from never smokers and light smokers had comparable TMB and silent to non-silent mutation ratio, suggesting these are similar entities. Additionally, in both subgroups, mutational signature 4 (associated with tobacco mutagens exposure) was absent, excluding second-hand tobacco exposure as a causative exposition in never smokers and suggesting that smoking does not significantly contribute to carcinogenesis in light smokers. On the other hand, these tumors were enriched in Signatures 16 (unknown) and 8, 3 (associated with DNA double-strand break repair by homologous recombination), 1 (spontaneous methyl-cytidine deamination associated with aging), and 2 and 13 (associated with activity of the cytidine deaminases AID and APOBEC). In this cohort, the most frequently mutated genes were *EGFR* (40%) and *TP53* (27%), and, interestingly, except for three tumors, all had amplification or mutation in *EGFR* or *MYC*. An ingenuity pathway analysis (IPA) was performed integrating data from mutated genes and CNAs and the affected pathways were related to cancer, epithelial cell adhesion and HER2 signaling (44).

A higher frequency of clinically actionable genomic alterations was detected among never smokers (N=160) in comparison to smokers (N=299) (65% vs. 35%, respectively). Samples were collected from patients coming from three American cancer institutions. Genomic data available from TCGA and from the Clinical Proteomic Tumor Analysis Consortium (CPTAC) was also analyzed. Institutional samples were submitted to WES (both in tumor and peripheral blood mononuclear cells) (N=88) or a Clinical Laboratory Improve Amendments (CLIA) validated targeted sequencing (N=17) and RNA sequencing of tumor samples from 69 patients. A model that incorporated TMB and mutation signatures characteristic of tobacco exposure was used to infer smoking status, apart from the self-reported smoking status. Never smokers showed a higher frequency of alterations in *EGFR, CTNNB1, SETD2, MET*, and *RB1.* The occurrence of pathogenic or likely pathogenic germline variants was similar between smokers and never smokers (6.4% vs. 6.9%, respectively), nevertheless mutations in cancer predisposition genes (*BRCA1, BRCA2, FANCG, FANCM, HMBS, MSH6, NF1, POLD1, TMEM127*, and *WRN*) were observed only among never smokers. Single base substitution (SBS) mutation signatures 1 (aging), and 2 and 13 (APOBEC) were seen in samples from never smokers as well, as described above, along with signature 6 (associated with mismatch repair deficient tumors). Interestingly, 5.9% of never smokers also showed smoking-related mutation signatures, suggesting second-hand smoking exposure (45).

**Figure 1:**
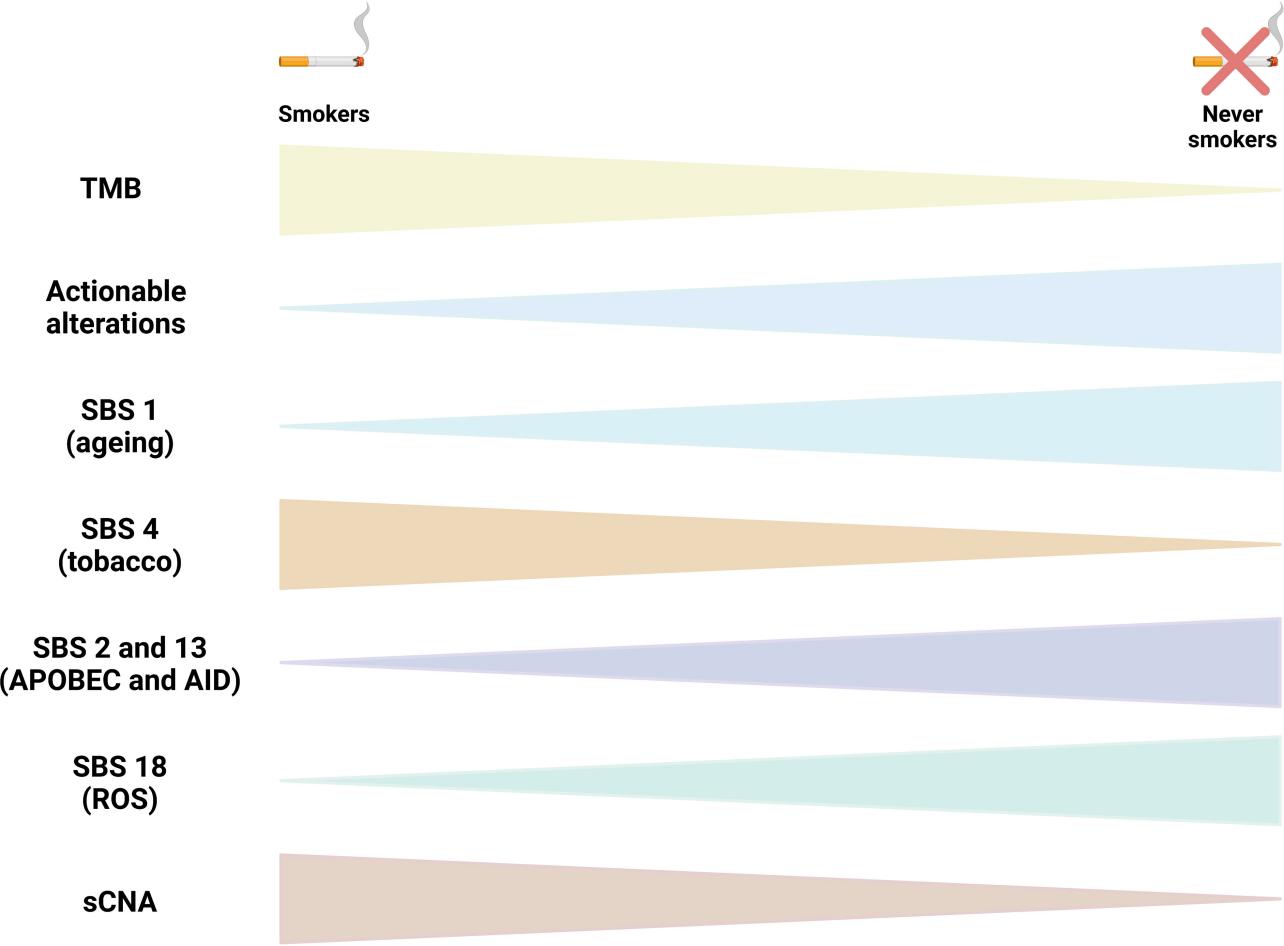
Molecular characteristics of lung cancer in non smokers (LCINS). The figure compile the recurrent molecular characteristics reported in studies that used whole exome sequencing (WES) and whole genome sequencing (WGS) to identify mutational processes end genomic characteristics in LCINS. Triangles tips point to the direction of the less frequent or less abundant alteration. TMB, tumor mutational burden; SBS, single base substitution mutational signature; sCNA, somatic copy number alterations; ROS, reactive oxygen species.

The largest genomic cohort comes from the Sherlock-Lung study that performed high-coverage WGS in tumor and matched germline DNA from 232 never smokers lung cancer patients. Alterations in genes in the RTK-Ras pathway were identified in 54.3% of tumors. These tumors had higher numbers of SNVs/indels, sCNAs, structural variants, kataegis, whole genome doubling and *BRCA2* loss of heterozygosity, but lower tumor/normal telomere length ratio. SBS 18, associated with damage by reactive oxygen species, was seen in 46% of tumors, SBS 2 and SBS13 (58%), and SBS8, related to nucleotide excision repair deficiency and late replication, was observed in 13%, mainly in carcinoids. Unsupervised clustering of arm-level sCNAs defined three subtypes with increasing levels of sCNA: piano, mezzo-piano and forte. Piano was the predominant subtype (49.6% of samples and the most common subtype among non-smokers) and was characterized by frequent *UBA1* mutations, germline *AR* variants, and stem cell-like properties like low TMB, high intratumor heterogeneity, long telomeres, frequent *KRAS* mutations and slow growth. In contrast, the mezzo-forte subtype (corresponding to 30.2%) was enriched in specific amplifications and *EGFR* mutations while the forte subtype was characterized by WGD. The Forte subtype represented 20.3% of samples and was enriched among smokers (18). The most recurrent molecular features reported in LCNIS are depicted in **Figure 1**.

## Immune landscape in never-smoker lung cancer patients

As discussed above, studies reveal that the genomic landscape in NSCLC is distinct between smokers and nonsmokers. NSCLCs from smokers have among the highest tumor mutational burdens (TMBs) of all cancers (46, 47). A high mutational smoking signature is associated with higher levels of immune infiltration, higher production of inflammatory cytokines such as IL-1beta and IL-17, higher cytolytic activity and interferon-gamma pathway signaling (48). In non-smokers, carcinogenesis is often linked to the presence of somatic molecular alterations in specific oncogenic drivers, like *EGFR* and *ALK* (49–51).

Immunosurveillance by host immune effectors imposes continuous selective pressure on tumor cells throughout the evolution of lung cancer, independent of the smoking status. Although both innate and adaptive immune responses are involved in antitumor immunity, host T cell recognition of tumor antigens represents the central tenet of immunosurveillance and immunoediting. Genomic mutations in lung cancer can give rise to mutant proteins that, when processed, result in the generation of neoantigens (52). CEA (carcinoembryonic antigen) is an oncofetal antigen produced during fetal life that disappears after birth. Oncofetal proteins reappear in some cancer patients, indicating that certain genes are reactivated during malignant transformation. Smokers have higher serum CEA levels than nonsmokers. CEA could serve as an ideal tumor-associated antigen (TAA), because immunizing cancer patients with TAA is expected to induce effective tumor immunity while not triggering serious autoimmune diseases (53).

In patients with early-stage NSCLC, investigators have identified CD8+ tumor infiltrating lymphocytes (TILs) that are reactive to tumor clonal neoantigens (50). Established lung cancers have escaped host immune surveillance through a variety of mechanisms. Tumors can directly suppress host immune responses by activating negative regulatory pathways known as immune checkpoints by tumor cells, such as expression of PD-L1. Lung tumors also restrict host immunosurveillance through suppression of functional antigen presentation. Lung tumors can also alter the composition of the TME to establish an immunosuppressive milieu characterized by an abundance of inhibitory molecules, such as TGF-beta, IL-6, PGE2 and VEGF, and an accumulation of immunosuppressive cells, such as Treg and MDSCs (47).

Differences in prognosis between smoking status groups may relate to the higher tumor immunogenicity, higher gene mutations, and more vigorous immune microenvironment observed in smokers. Results using CyTOF to characterize the immune TME found that the immune TME of lung cancer among smokers have higher expression of immune positive regulatory chemokines, and higher abundance of activated immune cells, including follicular helper CD4+ T cells, gamma delta CD4+ T cells, activated DC, and activated CD8+ T cells. In contrast, the immune microenvironment of tumor from the non-smoking group is enriched for immunosuppressive related cells, including regulatory T cells and M2 macrophages. Finally, the non-smoking group also contained higher fractions of CD45RAhigh CD4+ T cells and CD45RAhigh CD8+ T cells, typically characteristic of naïve T cell subpopulations (54).

Other evidence that the immune TME in non-smokers may be more suppressive than in smokers was seen examining infiltrating CD8+ T cells in non-smokers with lung adenocarcinoma. Potentially immunoregulatory CD8+FOXP3+ T cells and immune-dysfunctional CD8+GATA3+ T cells are increased in adenocarcinoma of non-smokers. CD4+FOXP3+ regulatory T cells expressing CCR4 and CCL17-expressing CD163+ M2-like macrophages also accumulated correlatively and significantly in adenocarcinoma of non-smokers. These immunosuppressive cells may promote tumor progression by creating a suppressive TME that inhibits effective anti-tumor responses in never-smokers with lung adenocarcinoma (55).

These characteristics may help explain why NSCLC adenocarcinomas in never smokers typically do not demonstrate durable responses with immune checkpoint blockade despite PD-L1 expression (smokers have a higher PD-L1 expression) (56). Interestingly, PD-L2 gene (PDCD1LG2) single nucleotide polymorphisms (SNPs) are associated with lung adenocarcinoma risk in female never-smokers, and 3 of these SNPs were negatively associated with PD-L2 expression in non-tumor tissue, but not in tumor tissue (57). Although PD-L1 expression has been shown to be associated with carcinogenesis, tumor differentiation and vascular invasion, the role of PD-L2 is still poorly explored. Mechanistically, at low antigen concentrations the interaction between PD-L2 and PD-1 inhibits strong B7-CD28 signals, while at high concentrations, the interaction between PD-L2 and PD-1 reduces cytokine production, but not T cell proliferation, but the correlation between these PDCD1LG2 SNPs and PD-L2 expression requires further investigation, which might provide further insight into the PD-1/PD-L2 axis in T cell function and lung carcinogenesis (57).

There are a series of other risk factors that may play an important role in the immune response amongst non-smoker lung cancer patients (58). In particular, air pollution and oxidative stress can lead to DNA damage, ROS release by resident lung macrophages and subsequent immunopathology and tissue damage *via* NF-kB and AP-1 pathways (59). Moreover, occupational exposure and inhalation of chemicals like silica and asbestos particles can result in an increase in collagen-producing fibroblasts and fibrous tissue remodeling surrounding the inhaled particles. This leads to reduced antitumor immunity due to the induction of macrophage and neutrophil infiltration into the lung tissue, resulting in an enhanced secretion of cytokines, chemokines and ROS (60). Despite the abundance of IL-12, silica dust causes FasL overexpression in lung tissue. Fas/FasL affects macrophage function, rendering them unable to activate neither themselves, nor other cells that are critical to recognize and remove malignant cells. Moreover, FasL overexpression due to silica can lead to an increased release of TNF-alpha and other proinflammatory cytokines. While this TME could lead to recruitment of new immune cells, an accompanying increase in TGF-beta production can result in a shift from an inflammatory function of FasL to a suppressive one (61). The inhalation of asbestos fibers can alter the function of NK cells and CD8+ cytotoxic T-lymphocytes leading to an impaired anti-tumor immune response and rendering these individuals at high risk for lung and pleural carcinogenesis (62).

Another serious risk factor for never-smoker lung cancer is pulmonary fibrosis. In this case, fibrosis of the lung originates from repeated and excessive connective tissue remodeling in response to recurring alveolar microinjuries. In these situations, fibroblasts respond to excessive and aberrant wound healing by entering hyperproliferation and change to a pro-fibrotic phenotype resistant to apoptosis. Activated fibroblasts are highly responsive to growth factors and cytokines, e.g. TGF-beta, connective tissue growth factor, platelet-derived growth factor and IL-6 (63, 64). This chronic low-level inflammatory process leads to an increased risk of lung carcinogenesis in patients with pulmonary fibrosis. Macrophages, as a source of proinflammatory and pro-fibrotic cytokines, have been linked to lung fibrogenesis as well (65), and neutrophils play a role in chronic inflammation in general (IL-8/CXCL8) (66). Lastly, pulmonary infections and the lung microbiome can modify the immune dynamics in lung cancer. Patients with a history of pulmonary infections caused by *Mycobacterium tuberculosis* (inflammation and fibrosis) have an increased risk of lung cancer (67).

A pathogenic lung microbiome may impact the likelihood of lung cancer, in smokers and non-smokers alike, contributing to tumor initiation and progression through production of bacteriotoxins and other proinflammatory factors (68), but more research is needed to decipher potentially beneficial vs. pathogenic microbiota with respect to lung cancer development and progression.

**Figure 2:**
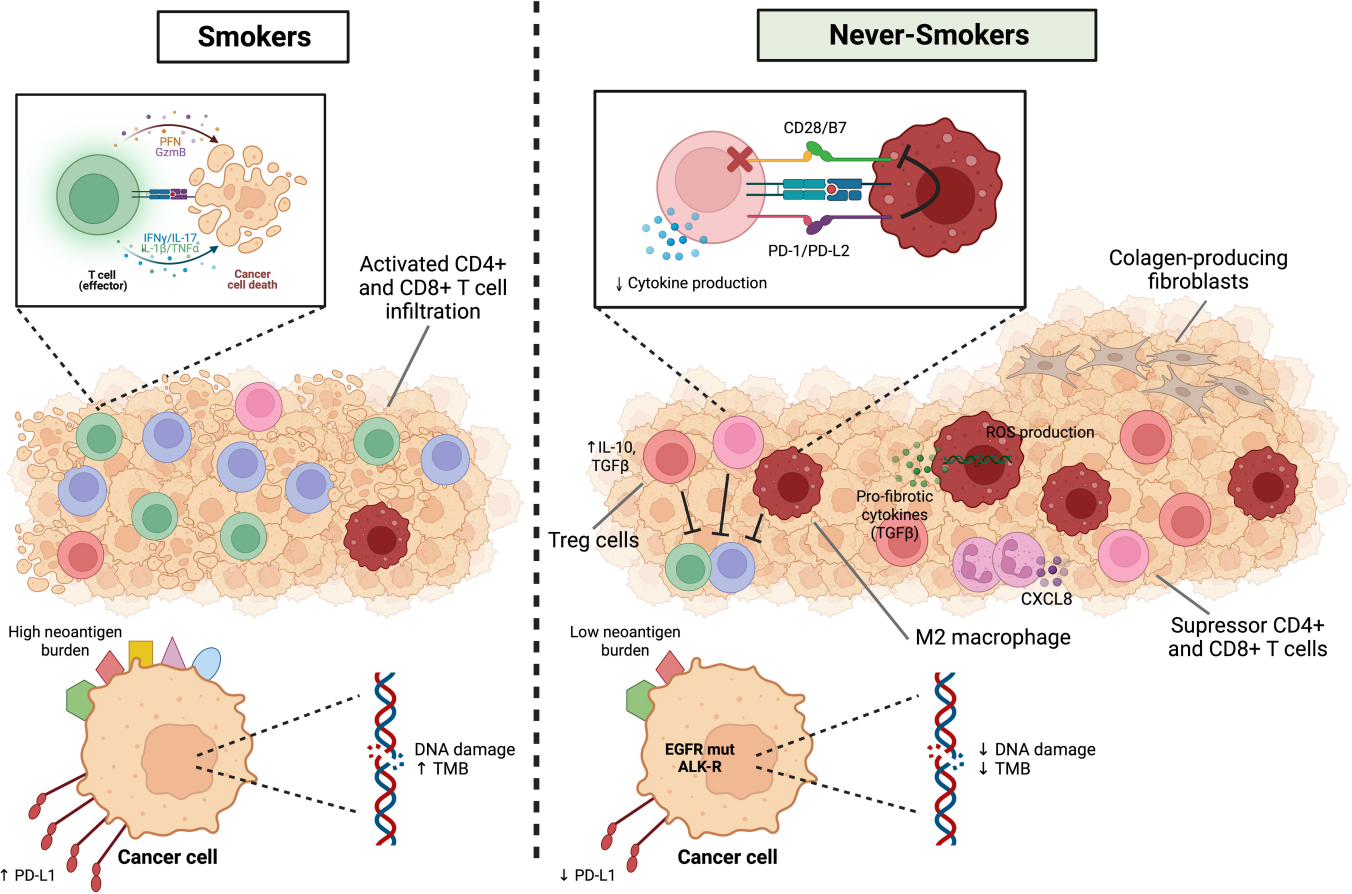
Immune landscape in smoker and never-smoker lung cancer patients. NSCLCs from smokers have high TMB and a higher mutational smoking signature and neoantigens generation are associated with higher levels of immune infiltration, higher production of inflammatory cytokines such as IL-1beta and IL-17, higher cytolytic activity and interferon-gamma pathway signaling. On the other hand, the immune microenvironment of the non-smoking group is significantly enriched for immunosuppressive related cells, including Treg cells, suppressor CD4+ or CD8+ T cells and M2 macrophages. In addition to the lower expression of PD-L1, the interaction between PD-L2 (APCs) and PD-1 (T cells) inhibits strong B7-CD28 signals and reduces cytokine production. In particular, air pollution and oxidative stress can lead to DNA damage and ROS release by resident lung macrophages and subsequent immunopathology and tissue damage. Macrophages can be a source of proinflammatory, and pro-fibrotic cytokines and neutrophils play a role in chronic inflammation in general (CXCL8). Finally, fibroblasts respond to excessive and aberrant wound healing by entering hyperproliferation and change to a pro-fibrotic phenotype resistant to apoptosis. Figure designed using Biorender.com.

In conclusion, the immune landscape of never-smoker lung cancer is influenced by several potential environmental factors that in general create a TME with potentially more immunosuppressive characteristics than smokers, and these contribute to a scenario where never-smoker patients may not respond as well to immune checkpoint inhibitors. **Figure 2** summarizes the immune landscape in smoker and never-smoker lung cancer patients. More research is necessary to unravel potential targets for activation of the immune response in these patients, or adjuvant therapy strategies that could make them more response to immune checkpoint inhibitors in the future.

## Clinical implications

### Immunotherapy for lung cancer in never smokers

Studies that investigated the use of immunotherapy (IO) in patients diagnosed with lung cancer without previous history of smoking have shown conflicting results, despite PD-L1 expression, and smoking history has been proposed as a biomarker associated with response to this therapy in NSCLC (69). Gainor et al. (2021) investigated the association between smoking history and the activity of immune checkpoint inhibitors in patients with metastatic NSCLC PD-L1 high-expressors. Outcomes were not significantly different among smokers (>10pack-years), light smokers (≤10pack-years) and never smokers (≤100 lifetime cigarettes), although a numerically smaller overall response rate (27% vs. 40% vs. 40%) and shorter progression free survival (3.0 vs. 4.0 vs. 5.4 months) and duration of response (6.9 vs. 10.8 vs. 17.8 months) were observed among smokers, never and light smokers, respectively (70). Similar results were reported by Popat et al. (2022) in a retrospective cohort of 1,160 patients treated with ICI monotherapy of which 91 patients were never smokers. Never smokers were mostly female, older and were diagnosed with non-squamous histology. OS was equivalent between ever smokers and never smokers (12.1 vs. 12.5 months, respectively). However, after adjusting OS for patient characteristics, OS among ever smokers was almost twice as long as amongst never smokers (12.8 vs. 6.5 months, respectively) (71).

Cortellini et al. (2021) (72) evaluated the outcomes of smokers versus never-smokers with advanced NSCLC and high PD-L1 ≥ 50% treated with pembrolizumab monotherapy in the first line in a retrospective multicenter cohort of 962 patients. They observed a higher risk of disease progression (hazard ratio [HR] = 1.49 [95% CI: 1.15–1.92], *p* = 0.0022) and death (HR = 1.38 [95% CI: 1.02–1.87], *p* = 0.0348) among never-smokers. A random case-control matching was performed and patients without a history of smoking maintained a higher risk of disease progression (HR = 1.68 [95% CI: 1.17–2.40], *p* = 0.0045) and a nonsignificant trend towards a reduced overall survival (OS) (HR = 1.32 [95% CI: 0.84–2.07], *p* = 0.2205).

Li et al. (2020) (69) performed a prospective real-world analysis of 268 patients with advanced NSCLC treated with anti-PD-1 monotherapy (IO) at the Princess Margaret Cancer Center and used logistic regression to test factors associated with response to treatment, including PD-L1 tumor proportion score (TPS) and smoking status. The cohort included 78 patients considered to be never smokers. They were more frequently female and had lung adenocarcinomas, 32% of tumors had *EGFR* mutations, 2.8% had *ALK* fusions, 58.3% had PD-L1 TPS ≥50%. Almost 90% of patients were treated with IO in ≥2nd line. Smokers or former smokers were predominantly of male sex and adenocarcinoma histology, without *EGFR/ALK* alterations, over 50% had PD-L1 TPS ≥ 50% and were also most commonly treated with IO as ≥ 2nd line (69.4% and 84.8%, for current and former smokers, respectively). Overall response rates (ORR) to immunotherapy were significantly higher in current and former smokers than never smokers (36% vs 26% vs 14%; p=0.02), even in patients with PD-L1 TPS ≥50% (current smokers 58% vs never-smokers 19%; p=0.03). One year survival rates, evaluated as an exploratory analysis, were also higher in smokers (p=0.003).

Another interesting fact is that never-smokers have a different genomic profile than smokers, with a higher prevalence of actionable driver gene alterations, such as *EGFR* mutations and *ALK* fusions, while *KRAS* mutations occur more often among smokers (73). Mazieres et al. (2019) (74) conducted a retrospective analysis of 551 patients with advanced NSCLC with at least one oncogenic driver alteration (*EGFR, KRAS, ALK, BRAF, ROS1, HER2, RET* or *MET*) treated with immune checkpoint inhibitor (ICI) monotherapy in 24 centers from 10 countries. All patients, except the ones with *KRAS, BRAF* or *MET* mutations, were more frequently never-smokers. The ORR by driver alteration was: *KRAS* 26%, *BRAF* 24%, *ROS1* 17%, *MET* 16%, *EGFR* 12%, *ERBB2* 7%, *RET* 6%, and *ALK* 0%. In the study, median PFS was 2.8 months, OS 13.3 months, and the best response rate 19%, mainly driven by the *KRAS* cohort. Overall, patients with actionable driver mutations (*EGFR, ALK, ROS1*) had inferior outcomes with ICI monotherapy, and PD-L1 expression did not appear to correlate with a better result in most cohorts.

Therefore, strategies to enhance response to ICI are urgently needed for LCINS, since the current strategies yield poor results. In cases where actionable driver gene mutations are detected, if available, targeted therapies should be exhausted before considering immunotherapy.

### Never smokers with actionable driver gene alterations and sequential immunotherapy

LCINS presents with a higher frequency of actionable driver gene alterations, such as EGFR and ALK, for which the use of tyrosine kinase inhibitors (TKI) has shown great benefit in several outcomes and should be the first choice of treatment (10). Nevertheless, many patients may also receive immunotherapy along the course of their disease, and there is great concern related to possible severe adverse events after sequential ICI and TKI (75). Schoenfeld et al. (2019) (76) performed a retrospective analysis of NSCLC patients with EGFR mutations who were treated with ICI and EGFR-TKI. 15% (6/41) of patients treated with ICI followed by Osimertinib presented with severe adverse events, which demanded corticosteroids use and, in some cases, hospitalization. The adverse events were more frequent among those who initiated Osimertinib within 3 months of prior ICI use. On the other hand, no patient treated with Osimertinib followed by ICI presented with severe adverse events. Lin et al. (2019) (75) identified 11 patients who had oncogenic alterations in ALK, ROS or MET and were treated with ICI followed by crizotinib. 45.5% (5/11) of patients presented with grades 3 or 4 increase in transaminases levels. All hepatotoxicity cases were reversible and non-fatal. Another example was the LIBRETTO-001 trial (77), in which NSCLC patients with RET mutations were treated with selpercatinib. The authors evaluated the frequency of severe adverse events among patients who were previously treated with ICI versus patients who were ICI-naïve. Among 329 patients, 22 presented with adverse events attributable to selpercatinib, which were more common in ICI-pretreated cohort (N = 17; 77%) than ICI-naïve cohort (N = 5, 23%).

Hence, the toxicity related to the sequential use of ICI and TKI is a great concern and should be carefully pondered before offered to NSCLC patients.

## Future perspectives

Future perspectives for treatment of LCINS with immunotherapy lie in enhancing immunologic activity, something that can be achieved by a myriad of different approaches. Currently, the majority of data is based, since trials that relied on smoking status as a surrogate biomarker for the presence of driver mutations, especially *EGFR*. By June 2022, there is only one trial recruiting non-smokers, and the population of this trial was mixed with *EGFR* mutant patients. **Table 1** shows data trials that evaluated exclusively (or at least mostly) LCINS from 2010 to 2022 on.

**Table 1:** List of clinical trials in non-small cell lung cancer that focused on non-smokers.

Trial	Design	Results	Status
NCT00456716 A Phase II Trial of Sorafenib in BAC or Never-Smokers With Any Lung Adenocarcinoma	Intervention: Sorafenib 400 mg BID. IC: any line of treatment, smoking history of < 100 cigarettes. 1EP: ORR.	N: 5 patients. Results: no results posted, study interrupted.	Completed. Last update posted March 4, 2010.
NCT00409006 A Phase 2 Trial of Pemetrexed and Cisplatin Followed Sequentially by Gefitinib Versus Pemetrexed and Cisplatin in Asian “Never Smoker” Patients With Advanced Non-Small Cell Lung Cancer	Intervention: Cisplatin + Pemetrexed + Gefitinib versus Cistplatin + Pemetrexed. IC: no previous treatment, smoking history of < 100 cigarettes. 1EP: PFS.	N: 70 patients (39 patients for Gefitinib + Chemo versus 31 patients for chemo alone). PFS: 9.9 months for Gefitinib + Chemo versus 6.8 months for Chemo.	Completed. Last update posted September 9, 2010.
NCT00430261 Phase II Trial of Sunitinib in Bronchoalveolar Carcinoma or Never-Smokers With Any Lung Adenocarcinoma	Intervention: Sunitinib 50 mg q42d (28 days on, 14 days of). IC: any line of treatment, smoking history of < 100 cigarettes. 1EP: PFS.	N: 20 patients. Results: no results posted, study interrupted.	Completed. Last update posted October 6, 2010.
NCT00455936 A Randomized Phase III Study of Gefitinib (IRESSATM) Versus Standard Chemotherapy (Gemcitabine Plus Cisplatin) as First-line Treatment for in Never Smokers Advance or Metastatic Adenocarcinoma of Lung	Intervention: Gefitinib 250 mg daily versus Cisplatin 80 mg/m2 D1 + Gemcitabine 1250 mg/m2 D1, D8 q21d. IC: no previous treatment, smoking history of < 100 cigarettes. 1EP: OS.	N: 315 patients. Results: no results posted.	Completed. Last update posted October 25, 2010.
NCT00550173 A Randomized Phase 2 Study Comparing Erlotinib-Pemetrexed, Pemetrexed Alone, and Erlotinib Alone, as Second-Line Treatment for Non-Smoker Patients With Locally Advanced or Metastatic Nonsquamous Non-Small Cell Lung Cancer	Intervention: Erlotinib 150 mg/daily versus Pemetrexed 500 mg/m2 q21d + Erlotinib 150 mg daily versus Pemetrexed 500 mg/m2 q21d. IC: second line of therapy, smoking history of < 100 cigarettes. 1EP: PFS.	N: 247 patients (81 for Erlotinib + Pemetrexed, 82 for Erlotinib, 84 for Pemetrexed). Results: PFS 7.4 months for Erlotinib + Pemetrexed, 3.8 months for Erlotinib, 4,4 months for Pemetrexed.	Completed. Last update posted February 13, 2013
NCT00976677 Randomized Double-Blind Placebo Controlled Phase II Trial Evaluating Erlotinib in Non-Smoking Patients With (Bevacizumab-Eligible and Ineligible) Advanced Non-Small Cell Lung Cancer (NSCLC)	Intervention: Carboplatin + Paclitaxel +/- Bevacizumab (Arm A) versus Carboplatin + Paclitaxel +/- Bevacizumab + Erlotinib (Arm B). IC: smoking history of < 100 cigarettes, previously untreated. 1EP: PFS.	N: 10 patients (5 for Arm A and 5 for Arm B). Results: PFS 4.5 months for Arm A versus PFS 15.5. More serious adverse events for Arm B – related to Erlotinib.	Completed. Last update posted May 30, 2014.
NCT01017874 A Randomized Ph 3 Study Comparing First-Line Pemetrexed/Cisplatin Followed by Gefitinib With Gefitinib Alone in East Asian Never Smoker or Light Ex-Smoker Patients With Locally Advanced or Metastatic Nonsquamous NSCLC	Intervention: Gefitinib 250 mg daily versus Cisplatin 75 mg/m2 D1 + Pemetrexed 500 mg/m2 D1 q21d + Gefitinib 250 mg daily. IC: no previous treatment, smoking history of < 100 cigarettes. 1EP: PFS.	N: 236 patients (118 in each group). Results: PFS 8.3 months for gefitinib + chemo versus 9.6 months for gefitinib alone. OS 26.9 months for gefitinib + chemo versus 27.9 months for gefitinib alone. No statistically significance difference found between groups.	Completed. Last update posted July 8, 2015.
NCT01404260 Intercalating and Maintenance Use of Iressa vs. Chemotherapy in Selected Advanced NSCLC: a Randomised Study	Intervention: continuous or intercalated Gefitinib + Carboplatin + Gemcitabine versus Carboplatin + Gemcitabine. IC: no previous treatment, smoking history of < 100 cigarettes or former light smokers (between > 100 cigarettes AND ≤ 10 pack-years AND quit ≥ 1 year ago). 1EP: PFS.	N: 219 (109 patients for Gefitinib + chemo and 110 patients for chemo alone). Results: PFS 9.7 months for Gefitinib + chemo versus 4.2 months for chemo alone.	Completed. Last update posted January 27, 2017.
NCT01344824 A Multicenter Phase II Trial of Carboplatin, Pemetrexed, and Bevacizumab Followed By Pemetrexed and Bevacizumab Maintenance Therapy in Patients With a Light or Never Smoking History	Intervention: Carboplatin + Pemetrexed + Bevacizumab + Erlotinib. IC: smoking history of < 100 cigarettes or former light smokers (between > 100 cigarettes AND ≤ 10 pack-years AND quit ≥ 1 year ago), previously untreated. 1EP: PFS.	N: 38 patients. Results: PFS 12.6 months. OS: 20.3 months.	Completed. Last update posted October 30, 2017.
NCT01829217 A Phase II Trial of Sunitinib in Never-smokers With Lung Adenocarcinoma: Identification of Oncogenic Alterations Underlying Sunitinib Sensitivity	Intervention: Sunitinib 50 mg q42d (28 days on, 14 days of). IC: one previous line of therapy, wild-type for KRAS, EGFR and ALK, smoking history of < 100 cigarettes, or RET positive tumors and other genomic alterations defined by protocol. 1EP: ORR.	N: 13 patients. Results: ORR 7.7%, no serious adverse events reported.	Completed. Last update posted October 31, 2018.
NCT00818441 A Phase 2, Open-Label Trial Of Dacomitinib (PF-00299804) In Selected Patients With Advanced Adenocarcinoma Of The Lung	Intervention: Dacomitinib IC: no previous treatment, EGFR mutant NSCLC or smoking history of < 100 cigarettes. 1EP: PFS in 4 months.	N: 89 patients. Results: PFS in 4 months 76.9%.	Completed. Last update posted January 8, 2019.
NCT00754923 A Phase II Study of Single Agent Sorafenib in Non-small Cell Lung Cancer Patients Who Never Smoked or Were Former Light Smokers.	Intervention: Sorafenib 400 mg BID. IC: at least 1 previous line of therapy, smoking history of < 100 cigarettes or former light smokers (between > 100 cigarettes AND ≤ 10 pack-years AND quit ≥ 1 year ago). 1EP: PFS in 6 months.	N: 11 patients. Results: PFS in 6 months: 0%. OS: 8.8 months.	Completed. Last update posted March 6, 2019.
NCT00445848 A Phase II Trial of the Combination of OSI-774 (Erlotinib; NSC-718781) and Bevacizumab (RHUMAB VEGF; NSC 704865) in Never-Smokers With Stage IIIB and IV Primary NSCLC Adenocarcinomas	Intervention: Erlotinib 150 mg/daily + Bevacizumabe 15 mg/kg q21d. IC: smoking history of < 100 cigarettes, previously treated. 1EP: OS.	N: 85 patients. Results: OS 29.8 months. PFS 7.4 months. ORR 50%. PD: 12%.	Completed. Last update posted: March 5, 2020.
NCT01833143 A Phase 2 Trial of Bortezomib in KRAS-Mutant Non-Small Cell Lung Cancer in Never Smokers or Those With KRAS G12D	Intervention: Bortezomib 1.3 mg/m2/dose D1, D4, D8, D11 q21d. IC: smoking history of < 100 cigarettes, KRAS G12D, previously treated NSCLC. 1EP: ORR.	N: 16 patients. Results: ORR 5.9%. SD 35.3%. PD 58.8%.	Completed. Last update posted: August 4, 2020.
NCT03786692 TH-138: Phase II Randomized Trial of Carboplatin + Pemetrexed + Bevacizumab, With or Without Atezolizumab in Stage IV Non-squamous NSCLC Patients Who Harbor a Sensitizing EGFR Mutation or Have Never Smoked	Intervention: Carboplatin + Pemetrexed + Bevacizumab + Atezolizumab (Arm A) versus Carboplatin + Pemetrexed + Bevacizumab (arm B). IC: chemotherapy, anti-VEGF therapy alone, and immunotherapy naive, EGFR mutant NSCLC or smoking history of < 100 cigarettes. 1EP: PFS.	N: 114 patients (estimated). Results: phase II, ongoing.	Recruiting. Last update posted February 10, 2022.

**Table 1**. Trials registered at ClinicalTrials.gov that aim as a primary population exclusively or mostly non-smokers. The majority of trials aim to use smoking status as a surrogate biomarker for EGFR positivity, considering that anti-EGFR therapies were included in combination with chemotherapy in 9 of the referred trials. Other strategies are mostly considering the role of antiangiogenic therapies, alone or in combination with chemotherapy. Only one trial is recruiting, and considers both EGFR mutant and non-smokers for treatment with Chemotherapy plus antiangiogenic therapy with or without anti-PD-L1 blockade.

*1EP, Primary Endpoint; IC, inclusion criteria; ORR, Overall Response Rate; N, Number of Patients; SD, Stable Disease; OS, Overall Survival; PFS, Progression Free Survival.

**Results and Clinical Trial data were obtained at www.clinicaltrials.gov by searching “Lung Cancer” as Condition and “Non-Smokers” as Other Terms. References otherwise non-specified relates to data obtained entirely from the www.clinicaltrials.gov website.

***Table was created by data obtained by online access at 27 Jun 2022.

### Combination therapy - chemotherapy, anti-PD-1/PD-L1 plus anti-CTLA4

As previously mentioned, the use of ICI monotherapy for LCINS has conflicting results in the literature and additional strategies to improve these results are in order. One possible approach is to associate chemotherapy with immunotherapy. In the KEYNOTE189 trial (78), patients with advanced nonsquamous NSCLC who were treated with platinum-based chemotherapy and pembrolizumab had a significant benefit in overall survival (OS), progression free survival (PFS) and ORR when compared to chemotherapy alone. Among the entire cohort, approximately 10% were never-smokers, and, in the subgroup analysis, these patients also benefited from the combination treatment, with an HR 0.23 (0.10-0.54 for OS). In the IMPOWER150 trial (79), which evaluated the benefit of adding atezolizumab to bevacizumab and platinum-based chemotherapy for advanced nonsquamous NSCLC, never-smokers, who represented approximately 20% of the cohort, had a trend towards better overall survival with the combination, with a median OS 22.3 months versus 18.2 months, HR 0.75 (0.49-1.14).

In the FDA pooled analysis of outcomes of anti-PD-(L)1 therapy (IO) with or without chemotherapy (chemo) for first line treatment of patients with NSCLC and PD-L1 score ≥50% (80), never-smokers appeared to have better results with the association of IO-chemo in all the outcomes evaluated: median OS NE vs 14.4 months (HR 0.39, 0.15-0.98), median PFS 10.2 vs 3.7 months (HR 0.46, 0.23-0.92) and ORR 69% vs 28% (OR 4.6, 1.5-14.5).

Combination of ICIs, especially of anti-PD-(L)1 and anti-CTLA4 antibodies, is another strategy currently available for prescription. However, results in non-smokers are based on subgroup analysis and are somewhat heterogeneous. The Checkmate227 trial (81) evaluated the benefit of nivolumab plus ipilimumab versus chemotherapy for patients with previously untreated advanced NSCLC. Although the study resulted in longer overall survival for the ICI combination in the intention to treat the population regardless of PD-L1 status, in the subgroup analysis, never-smokers, which represented 13% of the cohort, did not appear to derive benefit from this treatment approach, with an OS HR 1.23 (0.76-1.98). The Checkmate 9LA trial (82) investigated the benefit of nivolumab plus ipilimumab and two cycles of chemotherapy versus chemotherapy alone for patients with advanced NSCLC, and despite the benefit observed in overall survival for the entire cohort in the experimental arm, the subgroup analysis of never-smokers showed a trend towards better outcome with chemotherapy alone, with an OS HR 1.14 (0.66 - 1.97).

### Virotherapy

Recent studies have also been investigating the potential benefit of oncolytic virotherapy to improve response to immunotherapy in solid tumors (83). In a mouse model of small cell lung cancer, a modified oncolytic myxoma virus (MYXV) was associated with immune cell infiltration and increased survival, after low-dose cisplatin (84).

### Adoptive cell therapy and CAR-T-cells

Another possible way to overcome immunotherapy resistance in “cold” tumors is adoptive cell therapy (ACT), using tumor infiltrating lymphocytes (TIL) cultured from a patient’s tumor. In order to evaluate its benefit in patients with advanced NSCLC, Creelan et al. (2021) (85) conducted a single arm open-label phase I trial of TIL administered with nivolumab to 20 patients with advanced NSCLC who had initially progressed on nivolumab monotherapy. Thirteen patients had evaluable disease. Of these, three had confirmed responses, with 2 patients presenting complete responses ongoing 1.5 year later (one of which was a patient with lung adenocarcinoma and EGFR delExon19 mutation refractory to nivolumab). Eleven patients had reduction in tumor burden, with a median best change of 35%. In exploratory analysis, they found T cells recognizing multiple types of cancer mutations that were detected after TIL treatment, and were enriched in responding patients. The neoantigen-reactive T cell clonotypes increased and persisted in the peripheral blood after treatment, suggesting this strategy may be promising for patients with advanced lung cancer.

Modified-T-cell therapy, especially using chimeric antigen receptors (CAR-T cells) which can recognize specific molecular patterns on the surface of tumor cells, has been approved for the treatment of hematological malignancies and has been studied for solid tumors (86). Hu et al. (2020) (87) conducted a preclinical analysis of a CAR-T-cell based strategy to target specific neoantigens called LungX (s BPIFA1, PLUNC, and SPLUNC1) which are overexpressed in lung cancer cells. This strategy (called CARLunX) showed enhanced toxicity *in vitro* towards NSCLC lines and enhanced production of cytokines, as well as specific recognition of NSCLC cells. Adoptive transfer of these cells induced regression of established metastatic lung cancer xenografts and prolonged survival. This study suggested that CAR-T-cells could be a possible strategy to treat lung cancer patients and should be further analyzed, especially in never-smokers where recurrent molecular alterations are more frequently detected.

### Dendritic cells vaccines

Overall, studies have suggested that neoantigens, which are derived from tumor-specific somatic mutations, are related to responses to ICI therapy and ACT (88). There have been increasing efforts to identify these neoantigens in order to develop possible novel therapies, such as neoantigen-based RNA vaccines, which mainly use dendritic cells as vectors. Ding et al. (2021) performed a single-arm (88), pilot study, to evaluate the benefit of neoantigen vaccines for 12 patients with heavily pretreated advanced lung cancer. Four of these patients were never-smokers, three of which had EGFR or MET alterations. Candidate neoantigens were identified from whole-exome sequencing and RNA sequencing of fresh biopsy tissues as well as bioinformatics analysis, and 12–30 peptide-based neoantigens were selected for each patient. The ORR in this study was 25% and the disease control rate was 75%. Median progression free survival was 5.5 months and median OS was 7.9 months. In the study, four of the patients recruited had received ICI and either had no response to the treatment or had already relapsed. After combining the ICI therapy with the vaccines, all patients achieved disease control, with 2 partial responses of up to 80%, and showed a trend to better PFS (11.2 months versus 2.2 months, p = 0.045) and OS (11.2 months versus 7.6 months, p = 0.40). These results warrant further investigation of dendritic cell vaccines and may represent a therapeutic opportunity to improve ICI treatment results for lung cancer.

## Conclusion

Lung cancer in never smokers represent a different entity, with specific epidemiological features, genomic profile and tumor immune microenvironment. These patients more often present tumors that bear driver gene alterations potentially targeted by specific tyrosine kinase inhibitors. The majority of them, however, will be treated with immunotherapy as first or later lines of treatment, and the results of current trials in this specific population are still controversial. A better understanding of immunological features related to LCINS is paramount to aid the development of novel therapeutic strategies that may enhance immunotherapy responses in this subgroup of patients, and many are already under investigation.

## Author contributions

VA contributed with manuscript conceptualization and the writing of introduction, epidemiology and risk factors, Carcinogenesis and molecular characteristics of lung cancer in never smokers, clinical implications, future perspectives and conclusion sections, as well as revision and editing. AF contributed with the immune landscape in never-smoker lung cancer patients section and with **Figure 2**. MC contributed with the clinical implications and future perspectives sections and with **Table 1**. KG contributed with the immune landscape in never-smoker lung cancer patients section and reviewed the manuscript. VCCL contributed with manuscript conceptualization, writing carcinogenesis, molecular characteristics and clinical implication sections, elaboration of Figure 1 and manuscript revision and editing.

## Funding

KJG is a CNPq Research Fellow and his research is supported by the São Paulo Research Foundation (FAPESP) grant #2021/00408-6.

## Conflict of interest

The authors declare that the research was conducted in the absence of any commercial or financial relationships that could be construed as a potential conflict of interest.

## Publisher’s note

All claims expressed in this article are solely those of the authors and do not necessarily represent those of their affiliated organizations, or those of the publisher, the editors and the reviewers. Any product that may be evaluated in this article, or claim that may be made by its manufacturer, is not guaranteed or endorsed by the publisher.

## References

[1] Sung H Ferlay J Siegel RL Laversanne M Soerjomataram I Jemal A. Global cancer statistics 2020: GLOBOCAN estimates of incidence and mortality worldwide for 36 cancers in 185 countries. CA Cancer J Clin (2021) 71(3):209–49. doi: 10.3322/caac.21660 33538338

[2] Brownson R Alavanja M Caporaso N Simoes E CJ. Epidemiology and prevention of lung cancer in nonsmokers. Epidemiol Rev (1998) 20(2):218–36. doi: 10.1093/oxfordjournals.epirev.a017982 9919440

[3] Reitsma MB Kendrick PJ Ababneh E Abbafati C Abbasi-Kangevari M Abdoli A. Spatial, temporal, and demographic patterns in prevalence of smoking tobacco use and attributable disease burden in 204 countries and territories, 1990–2019: A systematic analysis from the global burden of disease study 2019. Lancet. (2021) 397(10292):2337–60. doi: 10.1016/S0140-6736(21)01169-7 PMC8223261 34051883

[4] IARC. Estimated number of new cases from 2020 to 2040, incidence, both sexes, age [0-85+] world. In: CANCERTOMORROW. IARC (2020). Available at: https://gco.iarc.fr/tomorrow/en/dataviz/tables?types=0&sexes=0&mode=cancer&group_populations=1&multiple_populations=1&multiple_cancers=1&cancers=39&populations=900.

[5] de Alencar VTL Formiga MN de Lima VCC. Inherited lung cancer: A review. Ecancermedicalscience. (2020) 14:1–13. doi: 10.3332/ecancer.2020.1008 PMC7039693 32104210

[6] Scagliotti G Longo M NS. Nonsmall cell lung cancer in never smokers. Curr Opin Oncol (2009) 21:99–104. doi: 10.1097/CCO.0b013e328321049e 19532009

[7] Smolle E Pichler M. Non-smoking-associated lung cancer: A distinct entity in terms of tumor biology, patient characteristics and impact of hereditary cancer predisposition. Cancers (Basel). (2019) 11(2):13–7. doi: 10.3390/cancers11020204 PMC6406530 30744199

[8] Casal-Mouriño A Valdés L Barros-Dios JM Ruano-Ravina A. Lung cancer survival among never smokers. Cancer Lett (2019) 451:142–9. doi: 10.1016/j.canlet.2019.02.047 30851418

[9] Toh CK Gao F Lim WT Leong SS Fong KW Yap SP. Never-smokers with lung cancer: Epidemiologic evidence of a distinct disease entity. J Clin Oncol (2006) 24(15):2245–51. doi: 10.1200/JCO.2005.04.8033 16710022

[10] Cho J Choi SM Lee J Lee CH Lee SM Kim DW. Proportion and clinical features of never-smokers with non-small cell lung cancer. Chin J Cancer. (2017) 36(1):1–7. doi: 10.1186/s40880-017-0187-6 28179026 PMC5299770

[11] Siegel DA Fedewa SA Henley SJ Pollack LA Jemal A. Proportion of never smokers among men and women with lung cancer in 7 US states. JAMA Oncol (2021) 7(2):302–4. doi: 10.1001/jamaoncol.2020.6362 PMC7716251 33270100

[12] Wakelee HA Chang ET Gomez SL Keegan TH Feskanich D Clarke CA. Lung cancer incidence in never smokers. J Clin Oncol (2007) 25(5):472–8. doi: 10.1200/JCO.2006.07.2983 PMC2764546 17290054

[13] Xufan L Wu J Zhou L Lu B Ying K Chen E. Smoker and non-smoker lung adenocarcinoma is characterized by distinct tumor immune microenvironments. Oncoimmunology (2018) 7(10):1–11. doi: 10.1080/2162402X.2018.1494677 PMC6169585 30288364

[14] Sun S Schiller J GA. Lung cancer in never smokers — a different disease. Nature (2007) 7(october):778–90. doi: 10.1038/nrc2190 17882278

[15] Corrales L Rosell R Cardona AF Martín C Zatarain-Barrón ZL Arrieta O. Lung cancer in never smokers: The role of different risk factors other than tobacco smoking. Crit Rev Oncol Hematol (2020) 148(September 2019):102895. doi: 10.1016/j.critrevonc.2020.102895 32062313

[16] Fontham ETH Correa P Chen VW Reynolds P Austin DF Williams A. Environmental tobacco smoke and lung cancer in nonsmoking women: A multicenter study. JAMA J Am Med Assoc (1994) 271(22):1752–9. doi: 10.1001/jama.1994.03510460044031 8196118

[17] Musolf A Simpson C Andrade M Mandal D Yang P Li Y. Familial lung Cancer: A brief history from the earliest work to the most recent studies. Genes (Basel). (2017) 8(36):1–13. doi: 10.3390/genes8010036 PMC5295030 28106732

[18] Zhang T Joubert P Ansari-Pour N Zhao W Hoang PH Lokanga R. Genomic and evolutionary classification of lung cancer in never smokers. Nat Genet (2021) 53(9):1348–59. doi: 10.1038/s41588-021-00920-0 PMC8432745 34493867

[19] Tomasek L. Lung cancer mortality among Czech uranium miners - 60years since exposure. J Radiol Prot (2012) 32(3):301–14. doi: 10.1088/0952-4746/32/3/301 22809823

[20] Jonsson H Bergdahl IA Åkerblom G Eriksson K Andersson K Kågström L. Lung cancer risk and radon exposure in a cohort of iron ore miners in malmberget, Sweden. Occup Environ Med (2010) 67(8):519–25. doi: 10.1136/oem.2009.047449 20647379

[21] Lane RSD Frost SE Howe GR Zablotska LB. Mortality (1950-1999) and cancer incidence (1969-1999) in the cohort of eldorado uranium workers. Radiat Res (2010) 174(6 A):773–85. doi: 10.1667/RR2237.1 21128801

[22] Neri A McNaughton C Momin B Puckett M Gallaway MS. Measuring public knowledge, attitudes, and behaviors related to radon to inform cancer control activities and practices. Indoor Air. (2018) 28(4):604–10. doi: 10.1111/ina.12468 PMC6047348 29704395

[23] Ferreccio C González C Milosavjlevic V Marshall G Sancha AM Smith AH. Lung cancer and arsenic concentrations in drinking water in Chile. Epidemiology. (2000) 11(6):673–9. doi: 10.1097/00001648-200011000-00010 11055628

[24] Alberg AJ Brock MV Samet JM. Epidemiology of lung cancer: Looking to the future. J Clin Oncol (2005) 23(14):3175–85. doi: 10.1200/JCO.2005.10.462 15886304

[25] Poinen-Rughooputh S Rughooputh MS Guo Y Rong Y Chen W. Occupational exposure to silica dust and risk of lung cancer: An updated meta-analysis of epidemiological studies. BMC Public Health (2016) 16(1):1–17. doi: 10.1186/s12889-016-3791-5 27814719 PMC5095988

[26] Brenner DR Hung RJ Tsao MS Shepherd FA Johnston MR Narod S. Lung cancer risk in never-smokers: A population-based case-control study of epidemiologic risk factors. BMC Cancer. (2010) 10 :1–9. doi: 10.1186/1471-2407-10-285 PMC2927994 20546590

[27] Loomis D Grosse Y Lauby-Secretan B El Ghissassi F Bouvard V Benbrahim-Tallaa L. The carcinogenicity of outdoor air pollution. Lancet Oncol (2013) 14(13):1262–3. doi: 10.1016/S1470-2045(13)70487-X 25035875

[28] Yap TA Ashok A Stoll J Mauer E Nepomuceno VM Blackwell KL. Prevalence of germline findings among tumors from cancer types lacking hereditary testing guidelines. JAMA Netw Open (2022) 5(5):e2213070. doi: 10.1001/jamanetworkopen.2022.13070 35594047 PMC9123503

[29] Jonsson S Thorsteinsdottir U Gudbjartsson DF Jonsson HH Kristjansson K Arnason S. Familial risk of lung carcinoma in the icelandic population. JAMA. (2004) 292:2977–83. doi: 10.1001/jama.292.24.2977 15613665

[30] Cannon-Albright LA Carr SR Akerley W. Population-based relative risks for lung cancer based on complete family history of lung cancer. J Thorac Oncol (2019) 14(7):1184–91. doi: 10.1016/j.jtho.2019.04.019 PMC6592768 31075544

[31] Amos CI Wu X Broderick P Gorlov IP Gu J Eisen T. Genome-wide association scan of tag SNPs identifies a susceptibility locus for lung cancer at 15q25.1. Nat Genet (2008) 40(5):616–22. doi: 10.1038/ng.109 PMC2713680 18385676

[32] Thorgeirsson TE Geller F Sulem P Rafnar T Wiste A Magnusson KP. A variant associated with nicotine dependence, lung cancer and peripheral arterial disease. Nature. (2008) 452(7187):638–42. doi: 10.1038/nature06846 PMC4539558 18385739

[33] Hung RJ Mckay JD Gaborieau V Boffetta P Hashibe M Zaridze D. A susceptibility locus for lung cancer maps to nicotinic acetylcholine receptor subunit genes on 15q25. Nature. (2008) 452:633–7. doi: 10.1038/nature06885 18385738

[34] Amos CI Pinney SM Li Y Kupert E Lee J de Andrade MA. A susceptibility locus on chromosome 6q greatly increases lung cancer risk among light and never smokers. Cancer re. (2010) 70(6):2359–68. doi: 10.1158/0008-5472.CAN-09-3096 PMC2855643 20215501

[35] Li Y Sheu CC Ye Y de Andrade M Wang L Chang SC. Genetic variants and risk of lung cancer in never smokers: A genome-wide association study. Lancet Oncol (2010) 11(4):321–30. doi: 10.1016/S1470-2045(10)70042-5 PMC2945218 20304703

[36] Parry EM Gable DL Stanley SE Khalil SE Antonescu V Florea L. Germline mutations in DNA repair genes in lung adenocarcinoma. J Thorac Oncol (2017) 12(11):1673–8. doi: 10.1016/j.jtho.2017.08.011 PMC5659909 28843361

[37] Oxnard G Heng J Chen R Koeller D Morgan R Wiesner G. Final report of the INHERIT EGFR study - 33 unrelated kindreds carrying germline EGFR mutations. J Thorac Oncol (2017) 12(11):S1758. doi: 10.1016/j.jtho.2017.09.355

[38] Hu X Yang D Li Y Li L Wang Y Chen P. Prevalence and clinical significance of pathogenic germline BRCA1/2 mutations in Chinese non-small cell lung cancer patients. Cancer Biol Med (2019) 16(3):556–64. doi: 10.20892/j.issn.2095-3941.2018.0506 PMC6743617 31565484

[39] Yamamoto H Higasa K Sakaguchi M. Novel germline mutation in the transmembrane domain of HER2 in familial lung adenocarcinomas. J Natl Cancer Inst (2013) 106(1):1–4. doi: 10.1093/jnci/djt338 PMC3906987 24317180

[40] Collisson EA Campbell JD Brooks AN Berger AH Lee W Chmielecki J. Comprehensive molecular profiling of lung adenocarcinoma: The cancer genome atlas research network. Nature. (2014) 511(7511):543–50. doi: 10.1038/nature13385 PMC4231481 25079552

[41] Ding L Getz G Wheeler DA Mardis ER McLellan MD Cibulskis K. Somatic mutations affect key pathways in lung adenocarcinoma. Nature. (2008) 455(7216):1069–75. doi: 10.1038/nature07423 PMC2694412 18948947

[42] Govindan R Ding L Griffith M Subramanian J Dees ND Kanchi KL. Genomic landscape of non-small cell lung cancer in smokers and never-smokers. Cell [Internet]. (2012) 150(6):1121–34. doi: 10.1016/j.cell.2012.08.024 PMC3656590 22980976

[43] Chen YJ Roumeliotis TI Chang YH Chen CT Han CL Lin MH. Proteogenomics of non-smoking lung cancer in East Asia delineates molecular signatures of pathogenesis and progression. Cell. (2020) 182(1):226–244.e17. doi: 10.1016/j.cell.2020.06.012 32649875

[44] Boeckx B Shahi RB Smeets D De Brakeleer S Decoster L Van Brussel T. The genomic landscape of nonsmall cell lung carcinoma in never smokers. Int J Cancer. (2020) 146(11):3207–18. doi: 10.1002/ijc.32797 31745979

[45] Devarakonda S Li Y Martins Rodrigues F Sankararaman S Kadara H Goparaju C. Genomic profiling of lung adenocarcinoma in never-smokers. J Clin Oncol (2021) 39(33):3747–58. doi: 10.1200/JCO.21.01691 PMC8601276 34591593

[46] Engels EA Wu X Gu J Dong Q Liu J Spitz MR. Systematic evaluation of genetic variants in the inflammation pathway and risk of lung cancer. Cancer Res (2007) 67(13):6520–7. doi: 10.1158/0008-5472.CAN-07-0370 17596594

[47] Salehi-Rad R Li R Paul MK Dubinett SM Liu B. The biology of lung cancer: Development of more effective methods for prevention, diagnosis, and treatment. Clin Chest Med (2020) 41(1):25–38. doi: 10.1016/j.ccm.2019.10.003 32008627

[48] Desrichard A Kuo F Chowell D Lee K-W Riaz N Wong RJ. Tobacco smoking-associated alterations in the immune microenvironment of squamous cell carcinomas. JNCI J Natl Cancer Inst (2018) 110(12):1386–92. doi: 10.1093/jnci/djy060 PMC6292793 29659925

[49] Dela Cruz CS Tanoue LT Matthay RA. Lung cancer: Epidemiology, etiology, and prevention. Clin Chest Med (2011) 32(4):605–44. doi: 10.1016/j.ccm.2011.09.001 PMC3864624 22054876

[50] Nicholas M Furness AJ Rachel R Sofie R Rikke L Kumar SS. Clonal neoantigens elicit T cell immunoreactivity and sensitivity to immune checkpoint blockade. Science (2016) 351(6280):1463–9. doi: 10.1126/science.aaf1490 PMC4984254 26940869

[51] Blons H Garinet S Laurent-Puig P Oudart JB. Molecular markers and prediction of response to immunotherapy in non-small cell lung cancer, an update. J Thorac Dis (2019) 11(Suppl 1):S25–36. doi: 10.21037/jtd.2018.12.48 PMC6353739 30775025

[52] Pirlog R Chiroi P Rusu I Jurj AM Budisan L Pop-Bica C. Cellular and molecular profiling of tumor microenvironment and early-stage lung cancer. Int J Mol Sci (2022) 23 :1–20. doi: 10.3390/ijms23105346 PMC9140615 35628157

[53] Nishimura Y Tomita Y Yuno A Yoshitake Y Shinohara M. Cancer immunotherapy using novel tumor-associated antigenic peptides identified by genome-wide cDNA microarray analyses. Cancer Sci (2015) 106(5):505–11. doi: 10.1111/cas.12650 PMC4452150 25726868

[54] Sun Y Yang Q Shen J Wei T Shen W Zhang N. The effect of smoking on the immune microenvironment and immunogenicity and its relationship with the prognosis of immune checkpoint inhibitors in non-small cell lung cancer. Front Cell Dev Biol (2021) 9:745859. doi: 10.3389/fcell.2021.745859 34660603 PMC8512705

[55] Kinoshita T Kudo-Saito C Muramatsu R Fujita T Saito M Nagumo H. Determination of poor prognostic immune features of tumour microenvironment in non-smoking patients with lung adenocarcinoma. Eur J Cancer (2017) 86:15–27. doi: 10.1016/j.ejca.2017.08.026 28950145

[56] Izumi M Sawa K Oyanagi J Noura I Fukui M Ogawa K. Tumor microenvironment disparity in multiple primary lung cancers: Impact of non-intrinsic factors, histological subtypes, and genetic aberrations. Transl Oncol (2021) 14(7):101102. doi: 10.1016/j.tranon.2021.101102 33930847 PMC8102176

[57] Liang S-K Chien L-H Chang G-C Tsai Y-H Su W-C Chen Y-M. Programmed death ligand 2 gene polymorphisms are associated with lung adenocarcinoma risk in female never-smokers. Front Oncol (2021) 11:753788. doi: 10.3389/fonc.2021.753788 34631591 PMC8497977

[58] Taucher E Mykoliuk I Lindenmann J Smolle-Juettner F-M. Implications of the immune landscape in COPD and lung cancer: Smoking versus other causes. Front Immunol (2022) 13:846605. doi: 10.3389/fimmu.2022.846605 35386685 PMC8978964

[59] Terzano C Di Stefano F Conti V Graziani E Petroianni A. Air pollution ultrafine particles: toxicity beyond the lung. Eur Rev Med Pharmacol Sci (2010) 14(10):809–21. 21222367

[60] Maeda M Nishimura Y Kumagai N Hayashi H Hatayama T Katoh M. Dysregulation of the immune system caused by silica and asbestos. J Immunotoxicol. (2010) 7(4):268–78. doi: 10.3109/1547691X.2010.512579 20849352

[61] Borges VM Falcão H Leite-Júnior JH Alvim L Teixeira GP Russo M. Fas ligand triggers pulmonary silicosis. J Exp Med (2001) 194(2):155–64. doi: 10.1084/jem.194.2.155 PMC2193452 11457890

[62] Nicholson WJ. The carcinogenicity of chrysotile asbestos–a review. Ind Health (2001) 39(2):57–64. doi: 10.2486/indhealth.39.57 11341559

[63] Fernandez IE Eickelberg O. The impact of TGF-β on lung fibrosis: from targeting to biomarkers. Proc Am Thorac Soc (2012) 9(3):111–6. doi: 10.1513/pats.201203-023AW 22802283

[64] Kolahian S Fernandez IE Eickelberg O Hartl D. Immune mechanisms in pulmonary fibrosis. Am J Respir Cell Mol Biol (2016) 55(3):309–22. doi: 10.1165/rcmb.2016-0121TR 27149613

[65] Murray PJ Wynn TA. Protective and pathogenic functions of macrophage subsets. Nat Rev Immunol (2011) 11(11):723–37. doi: 10.1038/nri3073 PMC3422549 21997792

[66] Obayashi Y Yamadori I Fujita J Yoshinouchi T Ueda N Takahara J. The role of neutrophils in the pathogenesis of idiopathic pulmonary fibrosis. Chest. (1997) 112(5):1338–43. doi: 10.1378/chest.112.5.1338 9367478

[67] O’Callaghan DS O’Donnell D O’Connell F O’Byrne KJ. The role of inflammation in the pathogenesis of non-small cell lung cancer. J Thorac Oncol Off Publ Int Assoc Study Lung Cancer. (2010) 5(12):2024–36. doi: 10.1097/jto.0b013e3181f387e4 21155185

[68] Kovaleva OV Romashin D Zborovskaya IB Davydov MM Shogenov MS Gratchev A. Human lung microbiome on the way to cancer. J Immunol Res (2019) 2019:1394191. doi: 10.1155/2019/1394191 31485458 PMC6710786

[69] Li JJN Karim K Sung M Le LW Lau SCM Sacher A. Tobacco exposure and immunotherapy response in PD-L1 positive lung cancer patients. Lung Cancer (2020) 150:159–63. doi: 10.1016/j.lungcan.2020.10.023 33171404

[70] Gainor JF Rizvi H Jimenez Aguilar E Skoulidis F Yeap BY Naidoo J. Clinical activity of programmed cell death 1 (PD-1) blockade in never, light, and heavy smokers with non-small-cell lung cancer and PD-L1 expression ≥50. Ann Oncol Off J Eur Soc Med Oncol (2020) 31(3):404–11. doi: 10.1016/j.annonc.2019.11.015 PMC7545963 32067682

[71] Popat S Liu SV Scheuer N Gupta A Hsu GG Ramagopalan SV. Association between smoking history and overall survival in patients receiving pembrolizumab for first-line treatment of advanced non–small cell lung cancer. JAMA Netw Open (2022) 5(5):e2214046–e2214046. doi: 10.1001/jamanetworkopen.2022.14046 35612853 PMC9133943

[72] Cortellini A De Giglio A Cannita K Cortinovis DL Cornelissen R Baldessari C. Smoking status during first-line immunotherapy and chemotherapy in NSCLC patients: A case-control matched analysis from a large multicenter study. Thorac cancer. (2021) 12(6):880–9. doi: 10.1111/1759-7714.13852 PMC7952794 33527756

[73] Cho J Choi SM Lee J Lee C-H Lee S-M Kim D-W. Proportion and clinical features of never-smokers with non-small cell lung cancer. Chin J Cancer. (2017) 36(1):20. doi: 10.1186/s40880-017-0187-6 28179026 PMC5299770

[74] Mazieres J Drilon A Lusque A Mhanna L Cortot AB Mezquita L. Immune checkpoint inhibitors for patients with advanced lung cancer and oncogenic driver alterations: results from the IMMUNOTARGET registry. Ann Oncol Off J Eur Soc Med Oncol (2019) 30(8):1321–8. doi: 10.1093/annonc/mdz167 PMC7389252 31125062

[75] Lin JJ Chin E Yeap BY Ferris LA Kamesan V Lennes IT. Increased hepatotoxicity associated with sequential immune checkpoint inhibitor and crizotinib therapy in patients with non-small cell lung cancer. J Thorac Oncol Off Publ Int Assoc Study Lung Cancer. (2019) 14(1):135–40. doi: 10.1016/j.jtho.2018.09.001 PMC6309637 30205166

[76] Schoenfeld AJ Arbour KC Rizvi H Iqbal AN Gadgeel SM Girshman J. Severe immune-related adverse events are common with sequential PD-(L)1 blockade and osimertinib. Ann Oncol Off J Eur Soc Med Oncol (2019) 30(5):839–44. doi: 10.1093/annonc/mdz077 PMC7360149 30847464

[77] McCoach CE Rolfo C Drilon A Lacouture M Besse B Goto K. Hypersensitivity reactions to selpercatinib treatment with or without prior immune checkpoint inhibitor therapy in patients with NSCLC in LIBRETTO-001. J Thorac Oncol Off Publ Int Assoc Study Lung Cancer. (2022) 17(6):768–78. doi: 10.1016/j.jtho.2022.02.004 PMC11083849 35183775

[78] Gandhi L Rodríguez-Abreu D Gadgeel S Esteban E Felip E De Angelis F. Pembrolizumab plus chemotherapy in metastatic non-Small-Cell lung cancer. N Engl J Med (2018) 378(22):2078–92. doi: 10.1056/NEJMoa1801005 29658856

[79] Socinski MA Nishio M Jotte RM Cappuzzo F Orlandi F Stroyakovskiy D. IMpower150 final overall survival analyses for atezolizumab plus bevacizumab and chemotherapy in first-line metastatic nonsquamous NSCLC. J Thorac Oncol Off Publ Int Assoc Study Lung Cancer. (2021) 16(11):1909–24. doi: 10.1016/j.jtho.2021.07.009 34311108

[80] Akinboro O Vallejo JJ Nakajima EC Ren Y Mishra-Kalyani PS Larkins EA. Outcomes of anti–PD-(L)1 therapy with or without chemotherapy (chemo) for first-line (1L) treatment of advanced non–small cell lung cancer (NSCLC) with PD-L1 score ≥ 50%: FDA pooled analysis. J Clin Oncol (2022) 40(16_suppl):9000. doi: 10.1200/JCO.2022.40.16_suppl.9000

[81] Hellmann MD Ciuleanu TE Pluzanski A Lee JS Otterson GA Audigier-Valette C. Nivolumab plus ipilimumab in lung cancer with a high tumor mutational burden. N Engl J Med (2018) 378(22):2093–104. doi: 10.1056/NEJMoa1801946 PMC7193684 29658845

[82] Paz-Ares L Ciuleanu T-E Cobo M Schenker M Zurawski B Menezes J. First-line nivolumab plus ipilimumab combined with two cycles of chemotherapy in patients with non-small-cell lung cancer (CheckMate 9LA): An international, randomised, open-label, phase 3 trial. Lancet Oncol (2021) 22(2):198–211. doi: 10.1016/S1470-2045(20)30641-0 33476593

[83] Ribas A Dummer R Puzanov I VanderWalde A Andtbacka RHI Michielin O. Oncolytic virotherapy promotes intratumoral T cell infiltration and improves anti-PD-1 immunotherapy. Cell. (2017) 170(6):1109–19. doi: 10.1016/j.cell.2017.08.027 PMC8034392 28886381

[84] Kellish P Shabashvili D Rahman MM Nawab A Guijarro MV Zhang M. Oncolytic virotherapy for small-cell lung cancer induces immune infiltration and prolongs survival. J Clin Invest. (2019) 129(6):2279–92. doi: 10.1172/JCI121323 PMC6546459 31033480

[85] Creelan BC Wang C Teer JK Toloza EM Yao J Kim S. Tumor-infiltrating lymphocyte treatment for anti-PD-1-resistant metastatic lung cancer: A phase 1 trial. Nat Med (2021) 27(8):1410–8. doi: 10.1038/s41591-021-01462-y PMC8509078 34385708

[86] Qu J Mei Q Chen L Zhou J. Chimeric antigen receptor (CAR)-t-cell therapy in non-small-cell lung cancer (NSCLC): Current status and future perspectives. Cancer Immunol Immunother. (2021) 70(3):619–31. doi: 10.1007/s00262-020-02735-0 PMC7907037 33025047

[87] Hu Z Zheng X Jiao D Zhou Y Sun R Wang B. LunX-CAR T cells as a targeted therapy for non-small cell lung cancer. Mol Ther oncolytics. (2020) 17:361–70. doi: 10.1016/j.omto.2020.04.008 PMC7210386 32405534

[88] Ding Z Li Q Zhang R Xie L Shu Y Gao S. Personalized neoantigen pulsed dendritic cell vaccine for advanced lung cancer. Signal Transduct Target Ther (2021) 6(1):26. doi: 10.1038/s41392-020-00448-5 33473101 PMC7817684

